# Negotiating Stakeholder Relationships in a Regional Circular Economy: Discourse Analysis of Multi-scalar Policies and Company Statements from the North of England

**DOI:** 10.1007/s43615-021-00143-9

**Published:** 2022-01-05

**Authors:** Aodhan Newsholme, Pauline Deutz, Julia Affolderbach, Rupert J. Baumgartner

**Affiliations:** 1 Geography, Geology and Environment, University of Hull, Hull, UK; 2 Spatial and Environmental Sciences, Trier University, Trier, Germany; 3 Institute of Systems Sciences, Innovation and Sustainability Research, University of Graz, Graz, Austria

**Keywords:** Circular economy, Resource efficiency, Critical discourse analysis, Supply chains, Regional development, England

## Abstract

Circular economy (CE) literature discusses the need for cooperation between different stakeholders to promote a CE; there is also an assumption regarding the benefits of loop closing on a local or regional scale. However, the potentially conflicting priorities, understandings, and expectations of the stakeholders involved have not been sufficiently addressed. Regional (or local) authorities have a responsibility to promote prosperity for stakeholders in their administrative region, within the constraints of national policy; conversely companies can have financial imperatives associated with stakeholders who may be globally distributed. Evidence of these conflicting priorities, the various positions stakeholder take, and their expectations of each other can be seen in the language choices regional actors make in their public-facing policy and report documents. The aim of the paper is to consider the challenges for creating a regional-scale CE that might arise from the differing priorities and values of companies and public agencies relating to specific places. It uses discourse analysis (including critical approaches) to examine how policy and business documents represent the stakeholders of the CE, their place in it, their priorities, and, importantly, the relationship between CE actors, focusing on the case of North Humberside on the North East coast of England. The plans set out in these reports are designed for external stakeholders and allow us to gain an insight into company and policy thinking in relation to CE developments in the coming years, including how they view each other’s roles. Findings indicate a shared motivation across scales and sectors for the CE as a means towards sustainable growth within which business plays a central role. However, there is a critical double disjuncture between different visions for implementation. First, between policy scales, a regional-scale CE is prioritised by regional policymakers, who have an interest in economic advantage being tied to a specific place and call for national scale support for their actions. Second, between regional policymakers and business, companies focus on their own internal operations and potential supply chain collaborations, with little attention given to the regional scale. This can be seen in the way organisations represent the actors of a nascent CE differently. In addition, a hegemonic business-focused growth discourse excludes other visions of the CE; the public are relegated to a passive role primarily as consumers and recipients of under-specified “opportunities” of wealth creation. CE theorisations need to incorporate and address these critical perspectives in order to support the development of strategies to overcome them.

## Introduction

The concept of a CE has rapidly gained popularity, evolving into a broad range of ideas which have taken prominence in policy and business discourses [1]. At heart a CE strives to reduce emissions, increase longevity of products, and close material loops of production, in order to minimise waste [2–5]; notably there are also more radical visions of CE as playing a role in an economy where growth is not the focus [6]. In this study, we support the definition of Geissdoerfer et al. [2] of the CE as a “regenerative system in which resource input and waste, emission, and energy leakage are minimised by slowing, closing, and narrowing material and energy loops” [p. 757]. Policy support for the CE includes the European Commission adopting its new CE Action Plan establishing CE thinking as a central strategy for the implementation of the European Green Deal in March 2020. The UK is also developing its own CE Package, building on the European policies [7]. At the national level in the UK, the Industrial Strategy [8], the Resource and Waste Strategy [9], and the Decarbonisation Strategy [10] all highlight the importance of the CE for the UK to meet its targets in relation to reducing carbon emissions and also to achieving a more sustainable and prosperous society for the years to come. Given that any potential transition to a CE is a new experience and involves many organisations and systems, developing an understanding both of how they view a CE, and what they consider their own and others’ roles, a new or adapted way of seeing key relationships and practices is needed. The policy and business discourse is where we see this new thinking develop.

Building a CE is widely recognised as involving collaboration between different stakeholders, with different motives and expectations for participating [11]. Cooperation across a supply chain, for example, requires consideration of the priorities of product designers, manufacturers, users, recovery/exchange, and disposal companies [2, 12, 13]. Although there has been a shift to identify responsibilities of companies to stakeholders beyond their shareholders [e.g. 14, 15], the motivations for business to pursue CE initiatives focus on new business opportunities improved financial return and efficiency savings [16]. There is an industry expectation of economic advantages from CE practices, which will be shared with value chain partners [17–19]. The relationship between the companies and the places where they operate, though, is seldom considered in this context. Taking a “place-based” approach to developing a CE, i.e. focusing efforts based around a particular location, has been a separate academic discussion with insufficient attention to companies and their supply chain relationships [20]. Furthermore, a place-based approach to a CE introduces additional stakeholders—involving not just relevant industry bodies, but also those representing the place itself, including local government and other public agencies [21–23]. These bodies can bring the additional motivation of seeking economic benefits for the place itself [24, 25].

Much of the discussion of the CE has overlooked the spatial dimension [26] focusing, for example on volume of material in resource recovery loops [e.g. 27] rather than geographic scale or location. Small-scale loop closing may draw on consumption slowing approaches to the CE such as repair which could create widely distributed demand for circular services [28, 29]. Attention to small-scale (i.e. local to regional) CE development has focused on place-based initiatives, [e.g., 30, 31] or regions [23]. Small scales of loop closing benefit from local contextual benefits, such as accessibility and connectivity between proximally located regional stakeholders [26], with the potential to implement new technology for resource recovery, albeit short rather than longer term agreements may be easier to achieve [23]. Notably, though, regional-scale initiatives are not independent of their national (and potentially supranational) context.

Developing a regional-scale CE implies harmonising the priorities of multi-scalar place-based and business interests, the implications of which have not been examined. The aim of this paper is to consider the challenges for creating a regional-scale CE that might arise from the differing priorities and values of companies and public agencies in the context of a specific place.

Drawing on the statements of policymakers and businesses, this paper employs a discourse analysis (DA) to better understand how significant CE stakeholders view both themselves and each other as part of the development of a regional CE. Additionally, we offer a multi-scalar approach to studying CE discourse in order to bridge the gap between various levels of governance and develop a nuanced regional perspective on CE development. Using North Humberside in the North East of England as our case study, we carried out a DA of both policy and business documents to assess how key stakeholders’ perspectives to the development of a regional CE differ with respect to:

- Motivations for the CE
- The importance of the region and the significance of places
- The identification of the key players and their roles for developing the CE especially at the regional scale.

This paper will next critically review literature on regional-scale CE and the application of DA to policy and business documents relating to the CE. The methods employed and the case study region are described in the “Multi-scalar Discourses and Circular Economy Approaches in North Humberside” section. The analysis of the policy and business documents studied is presented in the “Discussion” section with conclusions including future research directions and study limitations presented in the “Conclusions” section.

## Regional Collaboration for a Circular Economy

In this paper, we focus on research examining CE development in places (as opposed to production or consumption-oriented approaches) and in particular issues related to stakeholder perspectives. Regional-scale CE discussions are closely allied to, and sometimes explicitly engaging with, debates on industrial symbiosis (IS) [e.g. 23, 32, 33]. The place-based aspect of engagement between policymakers and business means that lessons from IS have resonance to debates about fostering CE initiatives at the regional scale, whether or not those initiatives are specifically IS-oriented.

IS can be described as the exchange of residues (waste and/or by-products) between companies [34], i.e. how pre-consumer waste loops can be closed at the early stage of the production system [35]. Moving from linear throughput to closed-loop material and energy use can reduce negative externalities associated with pollution and waste disposal, while also reducing demand for resources [34, 36–38]. IS networks, comprising multiple (potentially unrelated) bilateral transfers between companies, can extend over a range of scales from the local to the global [39]. However, IS is commonly associated with the local to regional scale [e.g. 34]. Geographic proximity between participating companies is seen as a supporting factor by drawing on relationships that already exist (e.g. in local business networks), facilitating the building of trust between stakeholders [22], aiding cost-effective implementation [24, 40], and enabling the identification of potential resource exchanges [26]. The present COVID-19-driven shift to online networking might serve as a test for the benefits of spatial proximity for face to face communications. Be that as it may, local- to regional-scale IS development has been and continues to be attempted as a deliberate policy initiative [23, 24].

The role of place-based authorities in IS has been the subject of debate with indications that, for example companies may respond better to a business-led approach [41] and that IS as an economic development initiative cannot override the constraints of geographic context [24]. Costa and Ferrão [21] advocated a “middle-out” approach whereby regional authorities assisted business in navigating the IS-supportive national policies (in Portugal), but which did not require direct policymaker engagement with IS. In the regional case of the Basque country in Spain, Rincón-Moreno et al. [42] emphasise the importance of local stakeholder collaboration for a CE, but with an assumption of common interests. Other recent work is seeking to build local coalitions and in particular is focusing on resource exchanges between local stakeholders [26]. IS research in the case of Finland emphasises the crucial role that individual “champions” play in organisations to facilitate IS initiatives [43] as they break down barriers between industrial sectors to improve collaboration opportunities. Other authors have utilised a social network analysis approach to review previous regional level IS research [44], with key barriers in relation to IS often associated with a lack of knowledge and technical experience to implement IS initiatives [45]. A common feature of these IS studies is social and economic barriers which remain when seeking to implement collaborative IS activities between diverse stakeholders.

The above body of work tends to be assuming an essentially common interest in IS, and more recently in the CE, so that the challenge is how to overcome barriers rather than a potentially more fundamental conflict of interest. Randles [46] examined the scalar mis-match between places and companies with plants in multiple locations globally—the company studied prioritised its internal (but geographically large scale) interests over those of the locations where it was based. Additionally, companies have supply chain connections that transcend countries, jurisdictions, and governance levels [47, 48]. The connection of companies to multiple regulatory jurisdictions can be problematic from an IS perspective [20], for example in terms of meeting a range of product specifications. CE initiatives will need to work within the constraints of these relationships [20] or else will involve a significant re-arrangement of practice. The spatial implications of this, especially in terms of place-based agencies’ ambitions to retain value locally, need consideration. Thus, this research steps back from specific barriers to or drivers for collaboration to examine the underlying perspectives of the stakeholders involved, using a multi-scalar policy context alongside businesses with a presence at the regional scale.

### Stakeholder Perspectives on Developing Circular Economy Activities

Given the collaborative effort and systemic changes needed to form a CE, understanding the views of those involved is an important basis for designing CE initiatives, not least in terms of who is considered to merit inclusion in the discussion [49]. Publicly accessible documents offer a means to assess how potential CE stakeholders are representing both themselves and each other. Furthermore, DA provides a means to look beneath the surface expression of texts to explore the intentions of authors that might not be explicitly stated [50, 51]. Previous research has taken this approach to studying policy in a number of sectors and settings [e.g. 52–54] and elucidating power dynamics in environmental politics [55].

Research applying DA to CE policy documents strongly suggests that policymakers are not emphasising the CE as a radical departure from current practice. Notwithstanding the rhetoric of the Green New Deal and ambitions of the EU CE package, EU CE policy continues to focus on end-of-life solutions [56], i.e. the CE is perceived as an approach to waste management rather than offering solutions for waste prevention. The CE is firmly situated as a sustainable growth policy at the EU [e.g., 57] and national scale, for example in Sweden [58], where CE policy is presented as a driver for competitiveness and job creation. Recent work on CE initiatives at the city scale in Europe has found that the interests of business actors and technological considerations are given more priority than citizens [59], with an emphasis on business competitiveness and policy perspectives [30, 58, 60] even when claims are made to being responsive to citizens [30, 59]. Farrelly and Seoane [61] explain that the public is often represented passively in the grammar of policy reports, suggesting they may not be fully involved in the process of policy framing.

Missing from previous CE research into stakeholder perspectives is a multi-scalar approach which examines the views of policymakers at different scales of each other looking not just at the regional scale, but considering that within the wider context. The authority responsible for decision-making by sub-national scales of government is both set and constrained by national policies. European CE policy formation goes through several rounds of consultations with stakeholders and is designed to set a strategy for the EU, while allowing for discretion within member states on how to implement action [62]. At the national level in England, governmental policy lays out a vision for the country in a particular respect; the proposals go through a consultation process to develop specific policy instruments [e.g. 63]. At the regional level in England, local authorities set an agenda for their region, although their autonomy is limited compared to European counterparts [64]. National government’s idea of empowering regions as actors in a policy field is not always matched by a requisite devolution of power [65], which has been indicated in fields such as climate governance, where regional-scale responsibilities are not necessarily matched by sufficient resources to tackle these issues locally [66].

From a business perspective, there are varied understandings of CE practices, and the term CE is often used interchangeably with sustainability in companies [67], with little systematic difference apparent between companies in Italy and the Netherlands. In Walker et al.’s [67] study, CE was portrayed by companies as a tool primarily to improve their environmental performance. Research in the UK indicates that business prioritises its own role in the development of a CE [49], somewhat dismissive of the public who are seen merely as consumers.

In this study, we draw on sustainability reports to gauge how companies position themselves with respect to the CE. Sustainability reports offer insights into a company’s practices in relation to societal concerns and sustainable development. Thus, they could serve as an indicator of intentions for implementing the CE, albeit the lack of a requirement or agreed template for CE matters in corporate sustainability reporting means that few companies directly disclose CE issues [68]. There is consequently a lack of consistency between companies but a tendency to focus on the prevention of waste. Work on sustainability reports more generally cautions that companies can use the disclosure of certain sustainability details to help generate favourable impressions of their sustainability performance, in turn preserving organisational legitimacy [69]. Springett [70] emphasised that businesses have actively sought to mitigate the radical edge of sustainable development so that it would merely refer to the level of environmental and social commitment that corporations are comfortable with. It is rare for a sustainability report to feature examples of how the business may be seeking to reduce output [70], for example. Other scholars argue that companies are not in actuality addressing sustainability issues but are merely creating an image of sustainability by paying lip service to the topic [71]. There is a risk that the business conceptualisation of sustainability can gain hegemony over the other interpretations, whereby important social and environmental issues could become side-lined [72]. With increasing urgency of sustainability issues, most notably climate change, in public debate, the intentions of business are highly significant. This paper builds on recent reporting research which finds that widely used guidelines for sustainability reporting are vague with regard to CE requirements and the inclusion of CE in reports is voluntary [68]. So now is the optimal time to conduct this study as it allows us to explore how companies present their CE activities when there are no formal requirements, and they have the freedom to choose what to include in their report.

In summary, the development of a CE requires the alignment of interests and a willingness to collaborate between different types of stakeholders who may operate across difference scales. Recent work has applied DA and critical discourse analysis (CDA) to CE policy documents at the EU and national scale on the one hand and to company documents on the other. Innovatively, this paper focuses on the regional scale, often deemed preferential for IS, within the national context. It utilises DA/CDA to compare multi-level policy documents with company reports, using a CE lens of analysis.

## Methods

In this paper, we are interested in studying the underlying meanings in policy and business documents; to do so, we will be using DA and CDA to uncover apparent stakeholders and their CE involvement at the regional level. The next section will firstly explain the reasoning for using DA and CDA in this study, and then an overview of the case study location will be given, followed by the theoretical framework used and the documents studied.

### Discourse Analysis and Critical Discourse Analysis Approach

Discourse can be defined as ideas, concepts, and categories through which meaning is given to phenomena and which is produced and reproduced through an identifiable set of practices [55]; thus, discourse has particular relevance to how social relations are discussed, and within which we include “place” as an item of analysis [73]. According to Hardy et al. [50] “discourse analysis provides a more profound interrogation of the precarious status of meaning” [p. 19], which allows the researcher to investigate the content of a text and elucidate alternative apparent meanings. The “discussion” itself is the object of analysis [74].

CDA allows the researcher to make connections between the language used in the text and the apparent meaning in social practice [75, 76] through its particular analytical concepts [65]. For example, the author of a business or policy report may have a clear agenda and intended audience, but CDA allows us to explore other apparent meanings and the approach to “social actors” (also known as participants in social practices) [77]. It places a strong focus on how different social actors are discussed and perceived, through the language used, which helps to elucidate the actors who are seen as key players and significantly those that are by implication excluded because they are not given a role or acknowledged in the discourse [77]. Thus, for example, we can gain an impression of how the stakeholders relevant to a regional CE are viewing both their own and each other’s role. The documents studied are of value as they offer insights into conceptual business/policy thinking in relation to CE-related topics. Moreover, substantial resources are given to producing these reports in organisations, demonstrating the value in studying their contents, in order to gain deeper understandings of organisational perspectives and how they externally communicate their CE performance.

We utilised an inductive approach to studying the reports. This ensured that wide-ranging CE-related topics were included in the analysis. Firstly, the full report was reviewed. Using an inductive approach, we recorded expressions relevant to CE themes (whether or not they included terms preconceived as relevant). Once the most pertinent CE-related topics were found, these sections of the report were revisited and studied in finer detail to conduct a comprehensive textual analysis in order to gauge the overall organisational perspective on this topic and how it relates to the wider message of the report. Noting that the CE is considered an “umbrella concept” [78], including diverse visions, while the business/policy reports themselves also varied in purpose and contents, therefore a flexible and agile approach was needed to select and analyse the most relevant CE material in each document. The aim was not to record the frequency of CE topics but to explore how the documents discussed concepts in relation to CE and how various social actors were portrayed at the regional level. We applied Van Leeuwen’s CDA approach to critically explore the wider context and how the CE is perceived, discussed, and manifested in various documents. Van Leeuwen’s approach to CDA focuses on linguistic features such as word choice and meaning and sociological aspects involving how these words are understood and interpreted in practice. By studying Van Leeuwen’s sociosemantic categories (see Table 1), we can bridge the gap between the literal and sociological representation of language in different social contexts [79], which helps to uncover the sociological meaning when examining specifically how participants of social practices are described in discourse [77].

**Table 1:** Sociosemantic categories for CDA and social actors (based on Van Leeuwen (2008))

CDA terms	Meaning
Personalisation vs functionalisation	Language used is emotional to connect on a personal level versus language which is practical and precise. Social actors can be represented by what they are (personalisation) versus by what they do (functionalisation)
Inclusion vs exclusion	Language which is purposefully inclusive using terms such as “we” or “us” versus language which is designed to leave out actors from the agenda. More broadly, social actors can either be included or left out of the document
Foregrounding vs backgrounding	Social actors can either be foregrounded with the use of emphasis or backgrounded by de-emphasising them
Genericization vs specification	Language used describes actors in a broad general sense or refers to particular identifiable individuals
Activation vs passivation	Language used to describe how actors are viewed, they are described as “agents” or “patients”, which suggests the level of importance of their role. Actors are either given an active or passive role in an activity

### Case Study Approach and Location

**Fig. 1:**
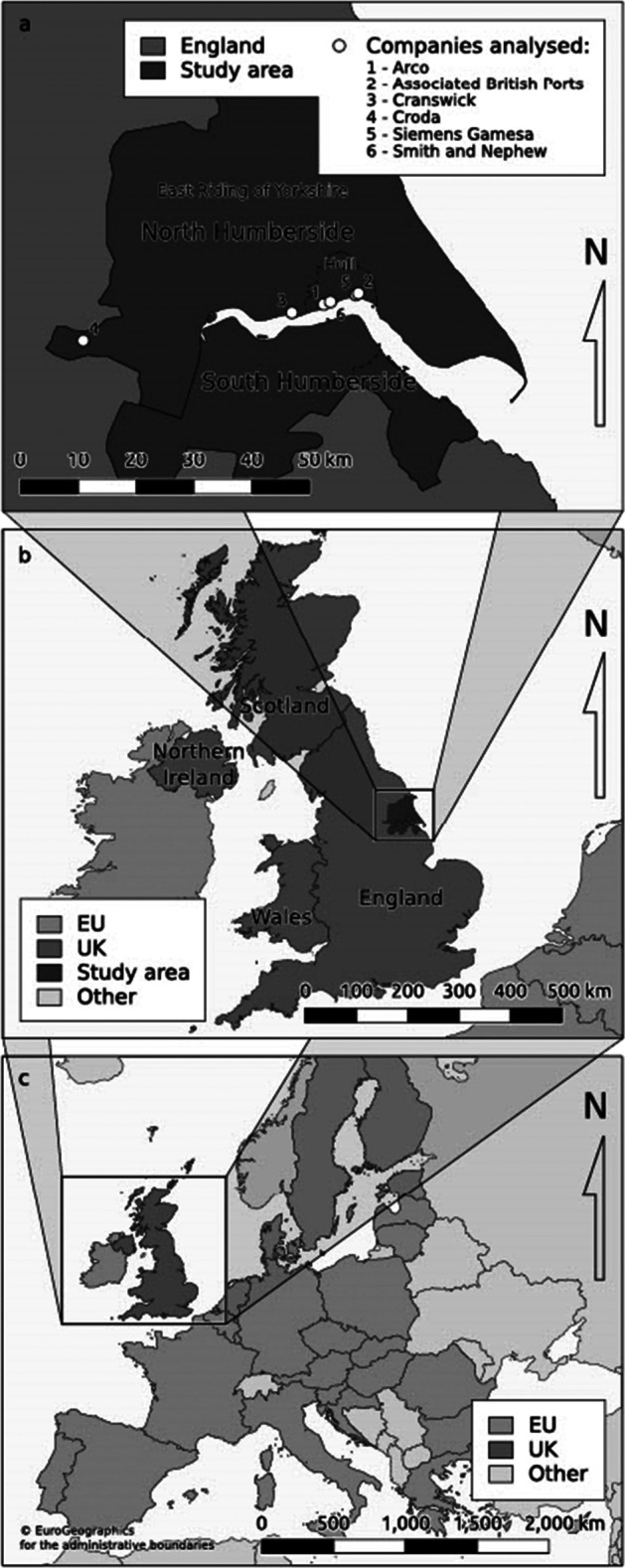
Case study location, North Humberside, England

We analyse CE activities with a particular focus on business and policy in North Humberside (Fig. 1), taking a multi-scalar and multi-sectoral case study approach ranging from the regional to national and EU scale. Although rooted in one place, and offering a contextualised insight to CE potential [80, 81], an in-depth case study can offer insights for other locations. We take a critical realist approach [82] which has been summarised in geographical terms as seeking “causal structures: particular combinations of contingent conditions and more general pressures that might explain the changing fortunes of particular places” [83, p.7]. In other words, through the lens of a particular place, we can uncover insights to processes and phenomena that are of wider relevance [84, 85].

North Humberside was chosen as it has a manufacturing-oriented economy, where both local policymakers and business appear to have a desire to shift towards a CE. The region comprises of the city of Hull and the East Riding of Yorkshire unitary authorities (combining the roles of district/metropolitan and county councils). Hull is a medium-sized city (estimated 260,673 inhabitants in 2017 [86]), and the East Riding is a largely rural authority with a population of 341,173 in 2019.^1^Predominant industries in the region are in manufacturing, chemicals, food, construction, and pharmaceutical, with the proximity to the Humber estuary and various ports being a key reason to set up in the region [87]. In the relatively deprived city of Hull, the manufacturing sector is one of the largest industries in the area with 54,000 people working in the wider engineering and manufacturing sectors [88]. The city boundaries of Hull are relatively small; many companies are located in the neighbouring and more prosperous East Riding of Yorkshire.

In addition to the local authorities, a further relevant public body is the Humber Local Enterprise Partnership (LEP). LEPs were created by the UK government in 2010 to drive economic development at a local level [89]. The Humber LEP uses the term “Energy Estuary” as a place-promotion technique which aims to shift from a heavily polluting region [90] to a cleaner, environmentally conscious economy (based in part on the offshore wind and bioenergy industry [91]). Previous academic research on IS has also focussed on the wider Humber region [e.g. 92–94], partially due to the industrial base of the region and in the past the presence of a regional “National Industrial Symbiosis Programme” office [94].

### Policy Documents

In total eleven policy reports were studied (Table 2). We identified reports to cover the scales of governance from an EU to the national English level and the regional level in Hull and East Riding (see Fig. 1). This offers an understanding of CE policy at the regional level in its multi-scalar context [64]. A transition to the CE is likely to rely on efforts of multi-actors across multiple scales [95]. Documents were chosen based on relevance to economic development, sustainability, and CE-related concepts. Notably, the policies in Table 2 are not legally binding but are designed to set an overarching strategy for their administrative area; hence, CDA is particularly useful due to the potential for diverging opinions and interpretations. Previous CE research has taken a similar multi-scalar approach within the EU/UK setting [e.g. 1]. Although EU policy is less directly relevant in the UK post-Brexit, it still acts as a benchmark when developing future environmental policy [7].

**Table 2:** Policy documents used in the analysis

EU level
[96]** European Industrial Strategy (2020)**
[97]** European Green Deal (2019)**
[98]** Circular Economy Action Plan: for a cleaner and more competitive Europe (2020)**
National level
[8]** UK Industrial Strategy: Building a Britain for the future (Department for Business, Energy and Industrial Strategy, 2017)**
[10]** UK Industrial Decarbonisation Strategy (Department for Business, Energy and Industrial Strategy, 2021)**
[7]** UK Circular Economy Package policy statement (Department for Environment, Food and Rural Affairs, 2020)**
[99]** Our Waste, Our Resources, a strategy for England (Department for Environment, Food and Rural Affairs, 2018)**
Hull and East Riding, Regional level
[90]** Humber Clean Growth Local White Paper (Humber LEP, 2019)**
[100]** Hull City Council Declaration of Climate Emergency (2019) and **[101]** Hull City Council 2030 Carbon Neutral Strategy (2020)**
[102]** East Riding of Yorkshire Council Economic Strategy (2018)**
[103]** East Riding of Yorkshire Council Environmental Policy (2017)**

### Business Documents

In total four reports and three websites from six companies were studied (Table 3). These companies were selected based on their presence in the case study region, their industrial sector, and the availability of sufficient quantity of data particularly related to sustainability issues. Thus, they are not self-selected by a willingness to participate or selected as representing CE best practice. Rather they represent what is present in the region, specifically in the manufacturing/logistics sector. Sustainability reports are publicly accessible documents that represent the company’s sustainability narrative to its community and customers, potential investors, and government bodies [104]. The companies selected have over 250 employees, with either the company headquarter or a subsidiary located in the region (see Table 3 for more company information). Smaller sized companies were not studied due to the lack of publicly available sustainability-related material. Based on these criteria, the companies studied represent the vast majority of manufacturing/logistics companies in North Humberside. Company reports were used to gain an insight into companies’ views on CE issues. We examined a number of the most recently available company sustainability reports to explore how they discuss sustainability topics, in particular CE issues. If sustainability reports were not available, we instead used company websites, which had dedicated sustainability/environmental sections.

**Table 3:** North Humberside, business documents

Company	Sector	Document	Location	Size and type
Siemens Gamesa	Wind-Turbine production	[105] Global Consolidated non-financial report 2019	UK production site in Hull	Large, 250 + employees, Public limited company
Cranswick	Food production sector	[106] National Annual Report 2019 and [107] Sustainability section on website	National Headquarter, East Riding of Yorkshire	Large, 250 + employees, Public limited company
Croda	Chemicals production	[108] National Sustainability report 2019	Global Headquarter, East Riding of Yorkshire	Large, 250 + employees, Public limited company
Arco	Workwear distribution	[109] Sustainability section on website	Global Headquarter in Hull	Large, 250 + employees, Family owned company
Smith and Nephew	Healthcare sector	[110] Global sustainability report 2019	UK production site in Hull	Large, 250 employees, Public limited company
Associated British Ports (ABP)	Port Authority	[111] Environment Section on Website	UK site in Hull	Large, 250 + employees, Public limited company

## Multi-scalar Discourses and Circular Economy Approaches in North Humberside

This section presents and analyses the findings with respect to the following themes: (1) “Motivations for the CE” based on the different ways CE is discussed in both policy and business reports; (2) “CE and spatial dimensions”, focused on different recognitions and expectations related to the role of the region and the scalar challenges when transitioning to a regional CE; and (3) “CE and key players”, which will explore how CE is discussed in terms of key social actors involved from both policymakers and business perspectives.

### Motivations for the CE

The analysis revealed several different expectations of a CE that are providing stakeholders with the motivation to consider CE-related activity, in addition to highlighting their views of each other.

From a policy perspective, EU, national, and regional level documents all address the role the CE plays in helping to achieve carbon neutrality while maintaining economic growth; “Circularity is an essential part of a wider transformation of industry towards climate-neutrality and long-term competitiveness” ([98] EU CE action plan, 2.3). Furthermore, use of language such as “competitiveness” suggests that the EU’s target audience is business; pursuing carbon neutrality is proposed as a long-term route to economic sustainability for business. The foregrounding of industry suggests that policymakers at the EU level see business as key agents of CE development in society.

At the national level, there is also evidence of resource efficiency and carbon neutrality being directly connected: “making new products from recycled materials (or secondary raw materials) can cause less harm, using less water and energy, and generating lower carbon emissions. When we create new markets for recycled materials, we also make recycling more economically viable”. ([99] Our Waste, Our Resources, 41). Businesses are excluded in the above quote, although it is apparent that policymakers are seeking to satisfy business concerns, by making resource efficiency activities economically viable. The reference to “making new products” suggests that national policymakers are implicitly speaking to the manufacturing industry. Besides carbon reductions, a further motivation is to secure material supply. This is typified by the following comment relating to England but is also found at the EU scale:

We will become more resilient to critical raw material shortages and less vulnerable to price volatility. A number of our initiatives will give businesses the confidence to invest more in resource-efficient technology and infrastructure, helping them to understand and mitigate risks in raw material supply chains and rewarding them for good product design. Importantly, society benefits too—experiencing all the rewards of a healthy, protected environment and a natural world that is being safeguarded from dangerous climate change. ([99] Our Waste, Our Resources, 25).

The first sentence in the above quote offers a personalised and inclusive approach to dealing with the issue of critical raw materials, which suggests all stakeholders share an interest and are expected to help address this issue. Evidently national policymakers are focussed on growth (i.e. ensuring materials supply) and thereby focusing on business priorities, which are postulated to benefit all stakeholders. The second sentence utilises a more functional approach and references “rewarding them” suggesting businesses are set to benefit in terms of financial performance by avoiding higher costs and backgrounding potential environmental benefits. The final sentence suggests that society is dealt with passively, and the public are patients of business/policymakers’ decisions when developing regional CE activities.

At the regional level, there is also an identified need for the Humber region to transition from a heavily polluting region to a location built on cleaner forms of production, and the CE is portrayed as a potential way to do so:

Hull, like the rest of the UK and the world, is at a cross roads in the journey from a place built on a fossil fuel economy to one built on renewable energy and production circularity. Our city has already chosen its direction through recent inward investment and its 2030 carbon neutral target. ([101] Hull City Council Carbon Neutral Strategy 2030, 10).

The mention of “a place built on fossil fuels” suggests passive, generalist, and functionalist language. This is identifying a need for change while not specifying either responsibly for the present circumstance or for changing it. There is acknowledgement of the need for cleaner forms of production in the Humber region, but this is indicated a shared problem (“like the rest of the world”). The city of Hull is heavily personified; reference to “Our city” illustrates both personalised and active language creating a sense of shared ownership, which may be designed to appeal to both local stakeholders (including the public) and prospective companies considering locating in the region*.* The council have foregrounded the role of business in achieving carbon neutrality by specifying “production circularity”, yet they also choose to exclude a direct reference to business in the quote.

From the business perspective too, CE is seen as a tool to help achieve carbon reduction targets and achieving cost reductions. This perspective is typified by this example: “Carbon management, waste reduction and improved resource efficiency remain key business priorities for ABP, with resource efficiency teams across the company looking at continual improvements that can be made” ([111] ABP website). The reference to “resource efficiency teams” appears to show an inward focus as they are solely discussing the potential of internal capacity in this respect. They cite “continual improvements that can be made”, which demonstrates the use of passive language, which creates a sense of sharing responsibility for implementation, while still implying they are taking action.

However, the majority of the attention in discourses is given to end-of-life CE issues in both policy and business strategies. At the national level, policy highlights the potential of the CE to improve resilience and secure resources as a support for competitiveness: “By using resources more efficiently we can ensure they are reused, re-manufactured or recycled as much as possible. Creating and safeguarding this stream of secondary resources will boost the resilience of UK businesses and enable them to become more competitive in the face of increasing and fluctuating commodity prices”. ([99] Our Waste, Our Resources, 120). The use of “we” demonstrates both personal and inclusive language suggesting all stakeholders need to participate. The next sentence offers more passive and functionalist language; “creating and safeguarding” resources elucidate the key role policy plays in order to enable business to become more resilient. The finer details of how these resources will be safeguarded are backgrounded in the discourse, while the actors needed to achieve these ambitions are excluded from the text.

End-of-life considerations are apparent in company reports. Arco’s CE initiatives appear to be motivated by end-of-life considerations, notably relating to packaging: “These include initiatives to make product packaging improvements as well as waste and energy management schemes. For example, 99% of packaging waste at the NDC is sent for recycling.” ([109] Arco website). The company addresses the need for internal resource efficiency initiatives, though these initiatives (such as waste management projects) are also likely to require collaborations with value chain partners. Functional language is used when discussing waste management, which is likely used to appeal to both suppliers and investors, as it gives them clear direction to plan future strategies. Arco appears to focus on end-of-life solutions, by placing a strong emphasis on recycling; however, it seems that recycling is outsourced, and in turn, the recycling organisation is backgrounded from the discussion. Arco also appear to distance themselves from the responsibility of their packaging waste; this is evident through the usage of the passive expression “sent for recycling”, which suggests they have not explored alternative CE strategies. Moreover, the company is a distributor located downstream in the supply chain, and they fail to acknowledge the potential for regional sourcing and collaboration opportunities for the CE.

However, CE narratives vary somewhat according to the nature of the company. For example, in sectors such as healthcare, environmental interests are less well tied to product development. For a healthcare product company: “The most critical requirement for packaging in healthcare applications is patient safety and ensuring product integrity. In addition to safety and economic considerations, we are also taking steps to improve the design of our packaging and ensure accurate tracking of packaging waste, to improve recovery and recycling rates.” ([110] Smith and Nephew, 33). This shows the company’s main priority is protecting product users and their health concerns, highlighting the need for high-quality packaging, which is prioritised above environmental concerns. They have foregrounded their own role in improving packaging standards, although there is likely a need for collaboration with external stakeholders. By specifying “product integrity” suggests the primary function of the packaging is to provide safety, which is paramount in the healthcare sector, so any CE ambitions must meet strict quality criteria. This mirrors the findings of Bodar et al. [112] in relation to hazardous substances and the challenges they create for CE activities.

The motivation for pursuing CE activities appears to be the possibility of combining environmental initiatives (carbon reduction and waste management) with economic initiatives such as competitiveness and material supply. Policy documents put more emphasis on material supply; business interests in the CE reflect company profile.

### CE and Spatial Dimensions

The following analysis will focus on how different social actors are discussed at different scales of governance in both policy and business reports, with particular attention given to the region of North Humberside.

In EU policy, there is explicit reference to scale (referred to as “level”) in terms of the societal challenges that a transition to a CE could cause: “The transition to the circular economy will be systemic, deep and transformative, in the EU and beyond. It will be disruptive at times, so it has to be fair. It will require an alignment and cooperation of all stakeholders at all levels—EU, national, regional and local, and international.” ([98] EU CE Action plan, 9). It is evident that the EU recognises the various scales that need to be involved, in order to implement CE initiatives. There is also acknowledgement that on the path to a CE, there may be disadvantages as well as benefits, notwithstanding the assumption of overall economic gain at the scale of the EU. This does raise a question as to the potential geography of disadvantage—i.e. might some places (be they cities, regions, or nations) be systematically disadvantaged; can the collaboration referred to counteract that or simply offset the effects? Moreover, the vague reference to the need for cooperation of “all stakeholders” generalises the role of different actors, suggesting that there is a lack of a clear CE implementation strategy for stakeholders at the regional level.

At the national English scale, there are examples of both spatially defined and non-spatially defined references. For example, the government sees the potential role for itself to act as a facilitator between both ends of the supply chain from the design stage to the recycling phase: “Facilitate better communication between recyclers and designers so that hazardous components are designed for easier dismantling, and destroyed to increase safe recycling operations” ([99] Our Waste, Our Resources, 119). Designers and recyclers are specified as key stakeholders and are given an active role in developing CE activities. There is no reference to place or scale—these supply/disposal chain relationships are not presented as possessing a spatial dimension or to involve a particular scale other perhaps that the implicit assumption that all parties are present within national boundaries. National policymakers also acknowledge their potential in bridging the gap between cross-sectoral partners to create CE collaborations:

One way to support businesses to innovate and achieve such savings is by supporting clusters of them to come together to share knowledge and good ideas with counterparts – this may be on a local or regional level, on a sectoral level, or by bringing two or more sectors together. By joining forces businesses can spread the costs and leverage the benefits from economies of scale. ([99] Our Waste, Our Resources, 44).

This quote recognises the need for economies of scales and cross sectoral collaboration between geographically proximal stakeholders to improve CE development across regions. Notably, the scale and form of potential collaboration is flexible—either spatially based at local to regional scale or a more metaphorical cluster between similar companies at the national scale. “By joining forces” offers a quasi-militaristic approach likely to create a sense of urgency; this vernacular and active language usage places a strong emphasis on the role of local business in tackling CE issues. Government appear to be calling on business to act, and policy appears willing to support; however, they rely on business to innovate and collaborate for the development of regionally focussed resource efficiency initiatives. National UK policymakers note the link between energy-intensive industries and deprived locations such as Humberside: “Many of the [existing high-energy] clusters are in relatively deprived regions and often act as a driver of prosperity for the surrounding area as key employers paying above the UK median wage” ([10] UK Industrial Decarbonisation Strategy, 122). Some caution is needed therefore that reducing carbon dependency does not increase regional inequalities.

At the regional scale, the CE is envisioned as a way to help the region, as distinct from and in addition to companies, to transition to cleaner forms of production while improving their economic performance: “The Humber could be at the heart of an offshore turbine circular economy industry which by 2030 will see the need to decommission around 750 MW of wind capacity and recycle more than 600 turbines each year – a huge economic opportunity” ([90] Humber Clean Growth Local White Paper, 16). This foregrounds the place itself and backgrounds the actors needed to do the work, albeit indicating the importance of business—portraying the “problem” of turbine decommissioning as an opportunity. Notwithstanding the prominence given to the relatively new (to the region) renewables sector, the council shares the national government’s concern for change in still carbon-intensive economy. However, the council is directly calling upon the national government to provide the enabling conditions to address local environmental issues. By implication the responsibility of regional stakeholders is backgrounded: “As Hull’s target is to achieve carbon neutrality by 2030, 20 years earlier than the national 2050 target, the Council, together with partners, will campaign for national policy changes and additional funding to ensure Hull’s carbon neutrality is delivered by 2030” ([101] Hull City Council Carbon Neutral Strategy 2030, 4). This suggests that the council sees the national government as holding the power when developing CE activities, as they are foregrounded in the carbon neutrality discussion. Regional policymakers believe more financial support is needed to tackle environmental issues locally, which suggests that there may be scalar misalignment between the views of national and regional government. However, it is clear that the LEP wants the benefits of the CE to remain locally: “Effective collaboration will transform the skills profile of the Humber, generating new and better paid jobs, maximising training resources and propelling the ambitions of individuals, business and the public and voluntary sectors” ([90] Humber Clean Growth Local White Paper, 25). Despite the challenges of shifting to a regional CE, especially in a heavily industrialised region like North Humberside, the LEP use broadly inclusive language to create a sense of togetherness between local actors and optimistically predict the potential for new job creation locally, while the EU heeds caution in relation to the disruptions which may occur in some locations. However, the social actors who need to collaborate are excluded from the discussion, potentially resulting in challenges for regional CE implementation.

From a business perspective, limited attention is given to the role of the region when developing CE initiatives. In general, business reports focus on optimising their supply chain systems, which is often done through collaborations across different locations, for example: “Siemens Gamesa has a strong history of supplier excellence, built up over the years through sustainable relationships with our supplier and contractor base” ([105] Siemens Gamesa, 68). The word “sustainable” here appears to suggests they have long-term agreements with these trusted suppliers. The company also references the importance of local suppliers and creating value for the local region: “Development of the local supply base adding highly technologically prepared and competitive competitors, while contributing to local wealth creation” ([105] Siemens Gamesa, 68). This implies that the wealth creation is shared by all stakeholders, which implicitly benefits the local community, although this is not explicitly claimed. The emphasis, however, is on traditional supply chain relationships rather CE collaborations. Furthermore, despite directly referring to the importance of local suppliers, the document goes on to highlight directly using the following Tier 1 suppliers in 2019: 11,340 suppliers in Europe, the Middle East, and Africa; 3,542 suppliers in Americas, and 3,571 suppliers in Asia/Australia (69). There seems to be disparity between their ambitions to source locally but their dependence on a globally distributed supply chain, which may create challenges for developing regional CE collaborations. Moreover, we see that Siemens Gamesa primarily discusses CE collaborations through supply chain partners who are actively considered in the document, rather than local connections.

### CE and Key Players

At the EU level, it appears that policymakers see member states and business as key enablers of CE initiatives: “Only a shared commitment from the EU, its Member States and regions, industry, SMEs and all other relevant stakeholders in a renewed partnership will allow Europe to make the most of the industrial transformation” ([96] EU Industrial Strategy, 6). The EU highlights the need for cross-collaboration between member states and local actors, but the role of the public is backgrounded as they are only included in the discourse if assumed to be one of “other relevant stakeholders”. There is direct reference to “a shared commitment” which announces the expectations of participation and highlights that actors are interdependent on one another; there is a perceived need for robust and trusting partnerships to achieve success. The EU perceives an economic potential of the CE through value chain collaboration: “It can deliver substantial material savings throughout value chains and production processes, generate extra value and unlock economic opportunities” ([98] EU CE Action Plan, 2.3). However, here the CE is personified as an actor (“it can deliver”); companies’ value chains and production processes are foregrounded in the discussion but as passive recipients of economic benefits. The reference to “material savings” and unlocking “extra value” effectively encapsulates the EU’s business-facing stance and ambitions for a CE, with an assumption of economic benefit within the EU.

At the national level, the government appears to be calling on business to act: “We are committed to moving towards a more circular economy – to raising productivity by using resources more efficiently, to increasing resilience by contributing to a healthier environment, and to supporting long-term growth by regenerating our natural capital” ([8] UK Industrial Strategy, 148). Similar to the EU approach, UK policy uses inclusive language such as “we are committed”, although businesses are implicitly cast as active players (e.g. needed to be more efficient in use of resources), without actually being mentioned in this statement. The CE is portrayed as a win–win scenario for both national government and business through a growth-centric narrative to economic development. The use of terms, such as “productivity” and “long-term growth”, also suggests that the government is seeking to appeal to companies, while the language used suggests that everyone will benefit from a transition to a CE. National government attempts to appeal to citizens to follow the CE strategy in order to build “…a healthier environment”. This reference to environmental health suggests the desire to connect with citizens offering a shared non-financial benefit to the CE.

Policymakers often refer to the importance of the public in developing CE initiatives, but they are usually passive recipients in the policy formation process. This can be seen in the following examples at the EU and national level, respectively: “Empowering consumers to play an active role in the circular economy, through better information on products and improved consumer rights” ([96] EU Industrial Strategy, 3.4) and “The role for communities throughout the country in driving productivity is a major component of our Industrial Strategy” ([8] UK Industrial Strategy, 168). Both at the EU and UK level, words such as “active” and “driving” foreground the role of the public by specifying community involvement, but in practice, communities are passive recipients of these CE activities, not involved in developing them. The role of the public at the EU scale is as consumers—i.e. to buy the more efficient products made by companies. A 2021 UK report which addresses decarbonisation discusses the potential role of CE activities such as reuse and repair. How this departure from a rigid growth orientation develops remains to be seen. It appears to provide the public increased responsibility in developing CE activities: “Increasing resource and material efficiency in practice means keeping products and materials in circulation for longer through circular economy approaches such as reuse, repair, recycling and reducing the quantity of materials used within manufacturing” ([10] UK Industrial Decarbonisation Strategy, 64). The finer details on implementing these CE activities are not specified; the generic language creates a level of ambiguity on both the role of the public and of these approaches to slowing consumption in CE implementation.

IS and resource efficiency collaborations between companies emerge in national and regional policy and business documents, again suggesting that industry is envisioned as a key actor in developing CE initiatives. National policy refers to IS opportunities in an informal style, potentially to appeal to business in a transparent and sympathetic way, by using personally oriented language. “Creating waste or by-products during manufacturing processes cannot always be avoided. But one company’s rejects can be another’s raw materials and we want to incentivise businesses to do just this.” ([99] Our Waste, Our Resources, 44). National policy recognises the need to adjust the playing field to incentivise IS activities between partner companies, but there is little recognition of the challenges and prerequisites needed to initiate and develop these collaborative relationships. The strategies needed to incentivise IS still appear to be in an early stage, but the region appears likely to be involved: “To begin with we will review the results being achieved in a small number of existing LEP-led, local authority or industry-led sectoral business clusters” ([99] Our Waste, Our Resources, 44). As with communities, though, the local authorities are conduits for strategies devised at the national scale.

At the regional scale, the LEP clearly sees the potential of CE activities for improving the prosperity of the region: “Industrial symbiosis, using the waste from one process as the raw material for another, is an opportunity to strengthen the Humber’s industrial cluster whilst contributing to the development of a circular economy that supports clean growth” ([90] Humber Clean Growth Local White Paper, 20). The direct reference to IS as an “opportunity” for the Humber illustrates the LEPs assumption of the potential economic and environmental benefits for the region of developing IS activities locally. This implies collaborations between local business actors, although the sentence refers to waste from a “process” rather than directly mentioning business. Industry is portrayed as benefiting. A role for local citizens is excluded from the agenda, at the regional level.

Business also recognise the opportunities of IS (without using the expression), as shown by this quote: “Prioritise materials that are re-used, re-manufactured, or recycled” ([110] Smith and Nephew, 9). This quote comes under the “Our sustainability vison and mission” section of their report which suggests that they have taken on an internal company perspective, but they are open to IS opportunities when sourcing inputs. The language used is general as it covers a broad range of issues but also functional and precise, which portrays their techno-centric approach to CE development. Similarly, ABP recognise they have unused materials which other companies may find useful as an input to their production process: “We aim to facilitate beneficial use opportunities for our dredged material as far as we can. We maintain a central register of our dredging and disposal operations.” ([111] ABP website). The use of language such as “facilitate” illustrates the potential role a well-placed port authority can play in fostering collaborative IS exchanges, but to “maintain a central register” appears to be a more passive approach. The complexities and challenges associated with passing materials between companies are not referred to.

Moreover, business reports share a sense that supply chain partners are key to companies and their CE journey. For example, Croda highlights life cycle assessments as being a key enabler of CE activities across their supply chain: “Increasing our Right First Time production rates, improving customer experience and reducing our carbon footprint. Conducting full life cycle assessments of our top 100 ingredients to help our customers to move towards a circular economy and reduce potential chemical hazards.” ([108] Croda, 103). They are located at an early stage in the supply chain and want their chemicals to be produced in a way that allows customers to close their loops of production downstream. The inclusion of “customer experience” suggests that both upstream and downstream value chain partnerships are key to circularity success at the company level. The role of external stakeholders is backgrounded, suggesting that the role of the regional actors is less central to their CE vision. Similarly, Cranswick’s CE ambitions are also portrayed through supply chain initiatives: “We will also support a UK circular economy by purchasing plastic trays with a minimum of 70 per cent recycled content” ([106] Cranswick, 35). This quote shows that Cranswick foreground national level collaboration in terms of CE implementation, while regional collaborations are not mentioned. Siemens Gamesa emphasise the need for increasing the recyclability of wind turbines, which is responding to an environmental handicap of what is by definition a product for reducing carbon emissions. The dual aims of economic and carbon advantage are closely entwined here: “Increasing the recyclability of turbine components is high on our agenda and we continuously take part in projects to support the development of a circular economy” ([105] Siemens Gamesa, 66). This quote foregrounds their own role in addressing change, the use of “we” and “our” emphasising their active role, albeit there is a suggestion of this as a future activity. The phrase “take part in” suggests collaboration with other actors, but who they are and what form they might take are unspecified; the company is representing its own willingness to engage with CE, while backgrounding those with whom it may need to collaborate to achieve its ambitions.

## Discussion

Documents from all the stakeholders studied identify the CE as a significant mechanism for reducing carbon emissions while providing opportunities for economic growth. The identification of the CE as primarily an end-of-life strategy, rather than a guide for re-imagining production and consumption, is also shared between them, notwithstanding references to remanufacturing, repair, and reuse as CE strategies primarily at the national scale. References are made to product design, but these are scarcer and less specific than end-of-life references. As observed by Springett [70], companies are keeping to comfortable ground, still not engaging with the environmental potential of design [113, 114]. Seemingly policymakers too are focusing on the relatively familiar and straightforward short-term measures, rather than seeking a more strategic approach to the CE with potentially more disruptive short-term implications. This may be self-defeating as the environmental benefits expected from the CE may not be fulfilled without more significant changes [115].

The spatial and scalar aspects of CE are under-defined in the discourses studied. The assumption of economic advantage from the CE is shared by all the documents, but the EU and national scale documents are silent on the spatial distribution of benefits within their territories. Those benefits tend to be assumed future “opportunities” for enhanced competitiveness rather than more immediate or tangible matters. For the EU and even at the national scale, there can be some expectation that the “disruptions” referred to by the EU are overall outweighed by the benefits—although this is unproven. Conversely at the regional scale, concrete benefits are needed, and the Humberside discourses studied indicate an expectation of benefit from the CE. The industries present provide both a need for change (given the carbon intensiveness of some of them) and potential for measures such as IS. However, the attraction of renewable manufacturing was supported by national and supranational policy. In that instance, Humberside was the geographic winner in a spatially competitive process [116]; but it is clearly not the only place seeking economic advantage from CE development [e.g. 25]. To take advantage of apparent possibilities, the regional discourse calls for more national government support including more funding to address regional issues. This echoes the findings of Farrelly [65] that the rhetoric and support from national government for regional action may diverge and also supports the findings of Vanhamäki et al. [23] in Finland. Thus, although local scale of governance might relish the potential of a local CE, but cannot make it happen without national scale support. Furthermore, capturing local advantage from the CE in the way outlined in these documents needs the cooperation of business.

However, while business sustainability reports indicate a willingness to be active in the CE, discourses are largely silent on potential regional CE initiatives. The companies foregrounded themselves as key players in CE implementation, in alignment with policy discourse at all scales. Notably, though the regional scale is backgrounded in business discussion along with mention of any particular location(s) (which is missing from the reports of companies closely tied to the region as well of those for whom Humberside is a relatively recent location). Rather than referring to collaboration with local public bodies, business discourse takes a firm business-centric view. Some companies discuss value chain partners including suppliers and customers as key actors when tackling sustainability issues. Other companies in this study regard CE initiatives as internal matters, seeing employee collaboration as key enablers of CE initiatives. Internal approaches to the CE may involve teams distributed across multiple locations, however. Potentially, companies are seeking to emphasise their own strengths and ability to take action [117] and therefore background the potential for engagement with other companies and stakeholders (as observed by Banerjee [118]). If this rhetoric of prioritising supply chain relationships and/or internal actions is translated into company behaviour, there are clear operational challenges to enabling regional CE collaborations. Thus, the business-centric approach to the CE may generate economic opportunities that are not necessarily based within a given place where a company may be located. Such approaches may or may not complement the visions of local-scale policymakers, who are not acknowledged as CE actors within the sustainability reports studied.

The language of the policy reports suggests not just that policymakers believe that industry is a key player in achieving CE targets, but also implies that policymakers accept a business/growth agenda for the CE. The public are decidedly backgrounded in, if not excluded from, the framing and implementation of a CE (as discussed in [30, 59] and more generally in policy terms by Farrelly and Seoane [61]). We note that the public’s role in these discourses is to benefit from environmental health and the (supposedly) shared benefits of wealth creation, which they help to generate by consuming more efficient products (and presumably in some cases by making them, though this is not mentioned). As with sustainability more generally, backgrounding can be a strategy to give business views “hegemony” over other stakeholder perspectives [119]. In the discourses studied, there is a strong identification of a growth narrative for the CE reinforced by both policy and business stakeholders. While not altogether surprising, the level of challenge to more radical visions of the CE is indicated by the recognition of business as a custodian of the CE. Additionally, ironically, there are significant issues for implementing a mainstream vision of the CE given that businesses needed for a regional CE do not include regional policymakers or the region as part of their framing of CE stakeholders.

## Conclusions

This paper has analysed place-based and company perspectives relating to the regional-scale development of a CE using the words of policymakers and business intended for public consumption. By considering both policy and company perspectives on a CE through the lens of a specific place, we have identified significant mis-matches of ambition, perceived power, and strategies for implementation of a CE. We refer to these mis-matches as a double disjuncture.

The first disjuncture relates to the contradictory positions between the different spatial policy scales—characterised by an expectation of regional-scale CE engagement but with local government identifying a shortfall in policy support from national government. Related to this, the region needs to ensure a specific benefit, whereas the policymakers operating at larger scales can be more content with the shared benefits assumed from a CE. At the regional level, the CE is not only discussed as a broad environmental and economic initiative but is more specifically envisioned as a way to enable the region successfully to transition from energy-intensive to cleaner forms of production. There is a tacit assumption that this region should be one of the economic beneficiaries of a transition to a CE, notwithstanding the ambiguity of the spatial distribution of the impacts of that transition.

The second disjuncture refers to differing understandings of policymakers and business relating to each others’ roles and relationship to places. Companies indeed are responding to ideas relating to a CE, but do not engage with the regional scale, or indeed acknowledge either the places where they are located or the multi-scalar policy contexts in which they operate. A commercial view is emphasised above spatial embeddedness. The business reports studied view CE opportunities through value chain collaborations or internal initiatives. However, they may be making a similar miscalculation as policymakers, by assuming that the collaborators they envisage (their suppliers and customers) would be willing to collaborate in CE initiatives. Power dynamics and contrasting priorities within individual supply chains may create complexities for collaborations, especially if collaboration may negatively impact the current economic performance of companies critical for the collaboration.

Thus, the double disjuncture is focused on the regional scale, where the expectations of policymakers are out of line with both the national policymakers and local business. Action from national government (or higher scale) is needed to tackle both sides of the disjuncture. This would involve support for local authorities as coalition-builders, given they are well-placed to develop links with and between business in their region. But equally important is setting a regulatory context for companies conducive to more transformative approaches to the CE, including incentivising the development of local connections. This could involve setting expectations relating to the CE within sustainability reporting. So far, however, policymakers and businesses have yet to make a break from the end-of-life approach to resource management that has evolved in the EU (and still strongly influences policy in England) over the last several decades.

Academic understandings of the CE and its potential are more ambitious compared to policymakers’ and business understandings. Theorisations of the CE need to engage with these critical discrepancies in order to help overcome the challenges indicated. This could better support the devising of transparent, cross-sectoral, and mutually beneficial cooperation between organisations that is necessary for the success of a regional CE and indeed the support/influence needed from other policy scales.

This study has some limitations. The analysis was focussed on policy and business reports, which may only provide a limited window into circular initiatives designed to appeal to certain audiences. Other research methods such as interviews with organisations could offer more nuanced CE understandings, compared to studying policy/business reports. Furthermore, conducting a similar study in a different geographic context may lead to new insights and help generate alternative perspectives on regional CE development.

## Data Availability

The data in this paper comes from the business reports/websites and policy reports studied, which are publicly available; full references to these websites/reports are provided in the reference list.

## Footnotes

1 https://intel-hub.eastriding.gov.uk/council-facts/
